# (*Z*)-3-(2-Chloro­benz­yl)-1,5-benzothia­zepin-4(5*H*)-one

**DOI:** 10.1107/S1600536812029030

**Published:** 2012-06-30

**Authors:** V. Sabari, R. Selvakumar, M. Bakthadoss, S. Aravindhan

**Affiliations:** a Department of Physics, Presidency College (Autonomous), Chennai 600 005, India; b Department of Organic Chemistry, University of Madras, Chennai 600 025, India

## Abstract

In the crystal structure of the title compound, C_16_H_12_ClNOS, the mol­ecules are linked into centrosymmetric *R*_2_^2^(8) dimers *via* pairs of N—H⋯O hydrogen bonds. The seven-membered ring adopts a boat conformation.

## Related literature

For the pharmaceutical properties of thia­zepin derivatives, see: Tomascovic *et al.* (2000 ▶); Rajsner *et al.* (1971 ▶); Metys *et al.* (1965 ▶). For conformations of thia­zepin derivatives, see: Huang *et al.* (2011 ▶). For graph-set analysis of hydrogen bonds, see: Bernstein *et al.* (1995 ▶).
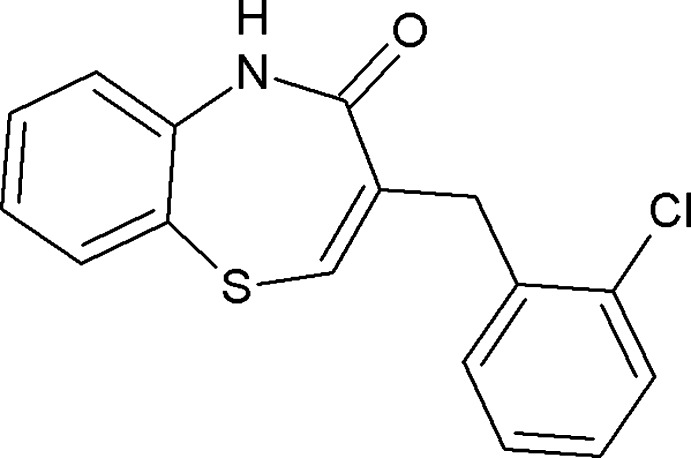


## Experimental

### 

#### Crystal data

C_16_H_12_ClNOS
*M* *_r_* = 301.78
Triclinic, 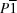

*a* = 8.4958 (3) Å
*b* = 8.7197 (3) Å
*c* = 10.0520 (3) Å
α = 101.930 (1)°
β = 95.179 (2)°
γ = 90.314 (2)°
*V* = 725.38 (4) Å^3^
*Z* = 2
Mo *K*α radiation
μ = 0.40 mm^−1^
*T* = 298 K
0.32 × 0.20 × 0.10 mm

#### Data collection

Bruker APEXII CCD area-detector diffractometer
Absorption correction: multi-scan (*SADABS*; Bruker, 2008 ▶) *T*_min_ = 0.980, *T*_max_ = 0.990
10314 measured reflections
3596 independent reflections
2961 reflections with *I* > 2σ(*I*)
*R*_int_ = 0.020

#### Refinement

*R*[*F* ^2^ > 2σ(*F* ^2^)] = 0.044
*wR*(*F* ^2^) = 0.129
*S* = 0.93
3596 reflections
185 parameters
H atoms treated by a mixture of independent and constrained refinement
Δρ_max_ = 0.51 e Å^−3^
Δρ_min_ = −0.54 e Å^−3^

### 

Data collection: *APEX2* (Bruker, 2008 ▶); cell refinement: *SAINT* (Bruker, 2008 ▶); data reduction: *SAINT*; program(s) used to solve structure: *SHELXS97* (Sheldrick, 2008 ▶); program(s) used to refine structure: *SHELXL97* (Sheldrick, 2008 ▶); molecular graphics: *ORTEP-3* (Farrugia, 1997 ▶); software used to prepare material for publication: *SHELXL97* and *PLATON* (Spek, 2009 ▶).

## Supplementary Material

Crystal structure: contains datablock(s) I, global. DOI: 10.1107/S1600536812029030/bt5944sup1.cif

Structure factors: contains datablock(s) I. DOI: 10.1107/S1600536812029030/bt5944Isup2.hkl

Supplementary material file. DOI: 10.1107/S1600536812029030/bt5944Isup3.cml

Additional supplementary materials:  crystallographic information; 3D view; checkCIF report

## Figures and Tables

**Table 1:** Hydrogen-bond geometry (Å, °)

*D*—H⋯*A*	*D*—H	H⋯*A*	*D*⋯*A*	*D*—H⋯*A*
N1—H1*A*⋯O1^i^	0.82 (2)	2.08 (2)	2.8911 (19)	174 (3)

Symmetry code: (i) 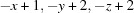.

**Fig. 1.**
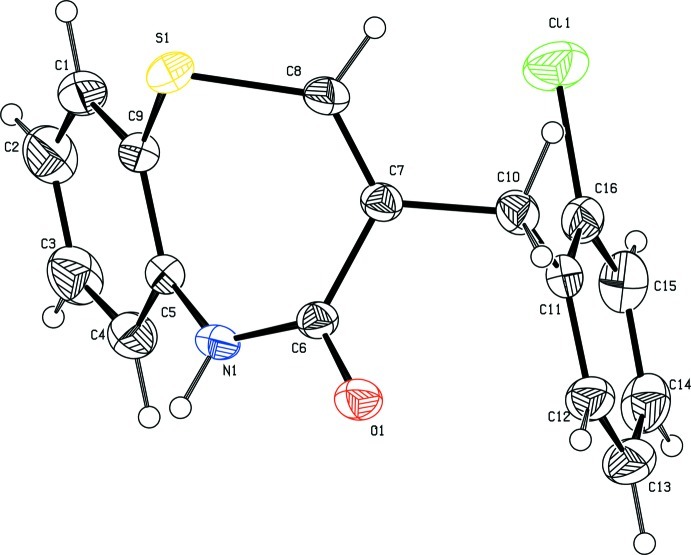
The molecular structure of the title compound, showing 30% probability displacement ellipsoids.

**Fig. 2.**
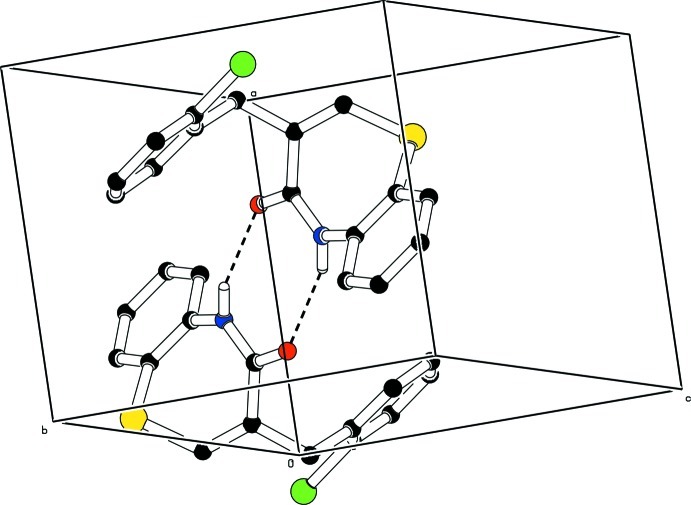
A view of the crystal packing. H atoms not involved in hydrogen bonding (dashed lines) have been omitted for clarity.

## supplementary crystallographic information

### Comment

The title compound is used as an intermediate for the synthesis of dosulepin, which is an antidepressant of the tricyclic family. Dosulepin prevents reabsorbing of serotonin and noradrenaline in the brain, helps to prolong the mood lightening effect of any released noradrenaline and serotonin, thus relieving depression. The dibenzo[c,e]thiazepin derivatives exhibit chiroptical properties (Tomascovic *et al.*, 2000). Dibenzo[b,e]thiazepin-5,5-dioxide derivatives possess antihistaminic and antiallergenic activities (Rajsner *et al.*, 1971). Benzene thiazepin derivatives are identified as a new type of effective antihistaminic compounds (Metys *et al.*, 1965). Considering the wide range of biological activities of the thiazepin derivatives,we determined the crystal structure of the title compound.

X-Ray analysis confirms the molecular structure and atom connectivity as illustrated in (Fig. 1). The seven membered thiazepin ring adopts a boat conformation (Huang *et al.*, 2011). The sum of the bond angles around the N1 atom (357.72°) indicates *sp*^2^ hybridization. The molecules are linked *via* N—H···O hydrogen bonds to centrosymmetric dimers with graph set notation *R*_2_^2^(8) (Bernstein *et al.*, 1995) (Fig.2).

### Experimental

A mixture of (*Z*)-methyl 2-(bromomethyl)-3-(2-chlorophenyl)acrylate (2 mmol) and *o*-aminothiophenol (2 mmol) in the presence of potassium *tert*-butoxide (4.8 mmol) in dry THF (10 ml) was stirred at room temperature for 1 h. After the completion of the reaction as indicated by TLC, the reaction mixture was concentrated and the resulting crude mass was diluted with water (20 ml) and extracted with ethyl acetate (3 *x* 20 ml). The organic layer was washed with brine (2 *x* 20 ml) and dried over anhydrous sodium sulfate. The organic layer was concentrated, which successfully provide the crude final product ((*Z*)-3-(2-chlorobenzyl)benzo[*b*][1,4]thiazepin-4(5*H*)-one). The final product was purified by column chromatography on silica gel to afford the title compound in 45% yields.

### Refinement

H atoms bonded to C were refined with fixed individual displacement parameters [U(H) = 1.2 *U*_eq_(C)] using a riding model with C—H ranging from 0.93 Å to 0.97 Å. The amino H atom was freely refined.

### Crystal data

**Table:**

C_16_H_12_ClNOS	*Z* = 2
*M**_r_* = 301.78	*F*(000) = 312
Triclinic, *P*1	*D*_x_ = 1.382 Mg m^−^^3^
Hall symbol: -P 1	Mo *K*α radiation, λ = 0.71073 Å
*a* = 8.4958 (3) Å	Cell parameters from 8725 reflections
*b* = 8.7197 (3) Å	θ = 2.8–29.1°
*c* = 10.0520 (3) Å	µ = 0.40 mm^−^^1^
α = 101.930 (1)°	*T* = 298 K
β = 95.179 (2)°	Triclinic, colourless
γ = 90.314 (2)°	0.32 × 0.20 × 0.10 mm
*V* = 725.38 (4) Å^3^	

### Data collection

**Table:**

Bruker APEXII CCD area-detector diffractometer	3596 independent reflections
Radiation source: fine-focus sealed tube	2961 reflections with *I* > 2σ(*I*)
Graphite monochromator	*R*_int_ = 0.020
Detector resolution: 15.9948 pixels mm^-1^	θ_max_ = 28.6°, θ_min_ = 2.1°
φ and ω scans	*h* = −11→11
Absorption correction: multi-scan (*SADABS*; Bruker, 2008)	*k* = −11→10
*T*_min_ = 0.980, *T*_max_ = 0.990	*l* = −12→13
10314 measured reflections	

### Refinement

**Table:**

Refinement on *F*^2^	Primary atom site location: structure-invariant direct methods
Least-squares matrix: full	Secondary atom site location: difference Fourier map
*R*[*F*^2^ > 2σ(*F*^2^)] = 0.044	Hydrogen site location: inferred from neighbouring sites
*wR*(*F*^2^) = 0.129	H atoms treated by a mixture of independent and constrained refinement
*S* = 0.93	*w* = 1/[σ^2^(*F*_o_^2^) + (0.0686*P*)^2^ + 0.3987*P*] where *P* = (*F*_o_^2^ + 2*F*_c_^2^)/3
3596 reflections	(Δ/σ)_max_ < 0.001
185 parameters	Δρ_max_ = 0.51 e Å^−^^3^
0 restraints	Δρ_min_ = −0.54 e Å^−^^3^

### Special details

**Table:**

Geometry. All e.s.d.'s (except the e.s.d. in the dihedral angle between two l.s. planes) are estimated using the full covariance matrix. The cell e.s.d.'s are taken into account individually in the estimation of e.s.d.'s in distances, angles and torsion angles; correlations between e.s.d.'s in cell parameters are only used when they are defined by crystal symmetry. An approximate (isotropic) treatment of cell e.s.d.'s is used for estimating e.s.d.'s involving l.s. planes.
Refinement. Refinement of *F*^2^ against ALL reflections. The weighted *R*-factor *wR* and goodness of fit *S* are based on *F*^2^, conventional *R*-factors *R* are based on *F*, with *F* set to zero for negative *F*^2^. The threshold expression of *F*^2^ > σ(*F*^2^) is used only for calculating *R*-factors(gt) *etc*. and is not relevant to the choice of reflections for refinement. *R*-factors based on *F*^2^ are statistically about twice as large as those based on *F*, and *R*- factors based on ALL data will be even larger.

### Fractional atomic coordinates and isotropic or equivalent isotropic displacement parameters (Å^2^)

**Table:**

	*x*	*y*	*z*	*U*_iso_*/*U*_eq_	
C1	0.3986 (3)	0.7811 (3)	0.4385 (2)	0.0611 (6)	
H1	0.3329	0.7850	0.3602	0.073*	
C2	0.5362 (3)	0.6996 (3)	0.4275 (3)	0.0727 (7)	
H2	0.5640	0.6500	0.3421	0.087*	
C3	0.6327 (3)	0.6915 (3)	0.5429 (3)	0.0686 (6)	
H3	0.7259	0.6362	0.5354	0.082*	
C4	0.5921 (2)	0.7650 (3)	0.6696 (2)	0.0554 (5)	
H4	0.6578	0.7587	0.7474	0.066*	
C5	0.45365 (19)	0.8482 (2)	0.68197 (17)	0.0400 (4)	
N1	0.42278 (17)	0.92803 (19)	0.81405 (14)	0.0425 (3)	
C6	0.29024 (18)	0.92977 (19)	0.87848 (16)	0.0352 (3)	
C7	0.13755 (18)	0.85995 (19)	0.80480 (17)	0.0361 (3)	
C8	0.0826 (2)	0.8806 (2)	0.68177 (19)	0.0455 (4)	
H8	−0.0194	0.8430	0.6497	0.055*	
C9	0.3563 (2)	0.8580 (2)	0.56553 (17)	0.0429 (4)	
C10	0.0416 (2)	0.7741 (2)	0.88754 (19)	0.0435 (4)	
H10A	0.0236	0.8440	0.9730	0.052*	
H10B	−0.0604	0.7426	0.8376	0.052*	
C11	0.12551 (19)	0.6308 (2)	0.91707 (17)	0.0382 (4)	
C12	0.2153 (2)	0.6381 (2)	1.0410 (2)	0.0506 (4)	
H12	0.2198	0.7312	1.1062	0.061*	
C13	0.2978 (3)	0.5119 (3)	1.0702 (2)	0.0615 (6)	
H13	0.3578	0.5212	1.1538	0.074*	
C14	0.2922 (3)	0.3731 (3)	0.9772 (3)	0.0622 (6)	
H14	0.3486	0.2882	0.9970	0.075*	
C15	0.2030 (3)	0.3594 (3)	0.8545 (2)	0.0594 (5)	
H15	0.1969	0.2646	0.7914	0.071*	
C16	0.1220 (2)	0.4874 (2)	0.82485 (18)	0.0466 (4)	
Cl1	0.01021 (9)	0.46470 (9)	0.66795 (6)	0.0789 (2)	
O1	0.29428 (15)	0.98862 (16)	1.00167 (12)	0.0457 (3)	
S1	0.18502 (6)	0.97237 (7)	0.57440 (5)	0.05265 (16)	
H1A	0.501 (3)	0.958 (3)	0.867 (2)	0.051 (6)*	

### Atomic displacement parameters (Å^2^)

**Table:**

	*U*^11^	*U*^22^	*U*^33^	*U*^12^	*U*^13^	*U*^23^
C1	0.0598 (12)	0.0839 (16)	0.0365 (9)	−0.0198 (11)	−0.0013 (8)	0.0081 (9)
C2	0.0641 (14)	0.0927 (18)	0.0539 (13)	−0.0154 (13)	0.0192 (11)	−0.0075 (12)
C3	0.0482 (11)	0.0836 (17)	0.0714 (15)	0.0037 (11)	0.0188 (10)	0.0043 (12)
C4	0.0397 (9)	0.0745 (14)	0.0536 (11)	0.0014 (9)	0.0038 (8)	0.0170 (10)
C5	0.0358 (8)	0.0482 (10)	0.0363 (8)	−0.0078 (7)	0.0001 (6)	0.0113 (7)
N1	0.0328 (7)	0.0581 (9)	0.0340 (7)	−0.0079 (6)	−0.0066 (6)	0.0076 (6)
C6	0.0359 (7)	0.0334 (8)	0.0362 (8)	−0.0005 (6)	−0.0041 (6)	0.0105 (6)
C7	0.0308 (7)	0.0366 (8)	0.0408 (8)	0.0011 (6)	−0.0018 (6)	0.0104 (6)
C8	0.0348 (8)	0.0536 (11)	0.0492 (10)	−0.0023 (7)	−0.0088 (7)	0.0186 (8)
C9	0.0420 (8)	0.0497 (10)	0.0373 (8)	−0.0107 (7)	−0.0029 (7)	0.0130 (7)
C10	0.0333 (8)	0.0494 (10)	0.0496 (10)	0.0012 (7)	0.0043 (7)	0.0139 (8)
C11	0.0347 (7)	0.0421 (9)	0.0396 (8)	−0.0047 (6)	0.0059 (6)	0.0118 (7)
C12	0.0593 (11)	0.0457 (10)	0.0452 (10)	−0.0068 (8)	−0.0056 (8)	0.0103 (8)
C13	0.0661 (13)	0.0602 (13)	0.0611 (13)	−0.0029 (10)	−0.0127 (10)	0.0279 (11)
C14	0.0671 (13)	0.0521 (12)	0.0759 (15)	0.0095 (10)	0.0129 (11)	0.0299 (11)
C15	0.0749 (14)	0.0442 (11)	0.0595 (12)	0.0017 (10)	0.0226 (11)	0.0051 (9)
C16	0.0504 (10)	0.0523 (11)	0.0373 (8)	−0.0065 (8)	0.0080 (7)	0.0082 (7)
Cl1	0.0987 (5)	0.0856 (5)	0.0433 (3)	−0.0106 (4)	−0.0118 (3)	0.0007 (3)
O1	0.0450 (7)	0.0514 (7)	0.0370 (6)	−0.0043 (5)	−0.0015 (5)	0.0029 (5)
S1	0.0507 (3)	0.0646 (3)	0.0485 (3)	0.0007 (2)	−0.0081 (2)	0.0308 (2)

### Geometric parameters (Å, º)

**Table:**

C1—C2	1.372 (4)	C8—S1	1.7554 (19)
C1—C9	1.393 (3)	C8—H8	0.9300
C1—H1	0.9300	C9—S1	1.767 (2)
C2—C3	1.373 (4)	C10—C11	1.510 (3)
C2—H2	0.9300	C10—H10A	0.9700
C3—C4	1.378 (3)	C10—H10B	0.9700
C3—H3	0.9300	C11—C12	1.390 (3)
C4—C5	1.387 (3)	C11—C16	1.393 (3)
C4—H4	0.9300	C12—C13	1.376 (3)
C5—C9	1.389 (2)	C12—H12	0.9300
C5—N1	1.414 (2)	C13—C14	1.366 (3)
N1—C6	1.347 (2)	C13—H13	0.9300
N1—H1A	0.81 (2)	C14—C15	1.371 (3)
C6—O1	1.236 (2)	C14—H14	0.9300
C6—C7	1.494 (2)	C15—C16	1.384 (3)
C7—C8	1.330 (2)	C15—H15	0.9300
C7—C10	1.513 (2)	C16—Cl1	1.740 (2)
			
C2—C1—C9	120.8 (2)	C5—C9—S1	121.50 (14)
C2—C1—H1	119.6	C1—C9—S1	119.38 (15)
C9—C1—H1	119.6	C11—C10—C7	111.24 (14)
C3—C2—C1	119.9 (2)	C11—C10—H10A	109.4
C3—C2—H2	120.0	C7—C10—H10A	109.4
C1—C2—H2	120.0	C11—C10—H10B	109.4
C2—C3—C4	120.2 (2)	C7—C10—H10B	109.4
C2—C3—H3	119.9	H10A—C10—H10B	108.0
C4—C3—H3	119.9	C12—C11—C16	116.03 (17)
C3—C4—C5	120.4 (2)	C12—C11—C10	120.21 (16)
C3—C4—H4	119.8	C16—C11—C10	123.74 (16)
C5—C4—H4	119.8	C13—C12—C11	122.1 (2)
C4—C5—C9	119.62 (17)	C13—C12—H12	118.9
C4—C5—N1	117.68 (16)	C11—C12—H12	118.9
C9—C5—N1	122.60 (17)	C14—C13—C12	120.3 (2)
C6—N1—C5	129.94 (14)	C14—C13—H13	119.8
C6—N1—H1A	112.3 (15)	C12—C13—H13	119.8
C5—N1—H1A	115.5 (16)	C13—C14—C15	119.7 (2)
O1—C6—N1	119.72 (14)	C13—C14—H14	120.2
O1—C6—C7	118.76 (15)	C15—C14—H14	120.2
N1—C6—C7	121.52 (14)	C14—C15—C16	119.7 (2)
C8—C7—C6	123.87 (16)	C14—C15—H15	120.1
C8—C7—C10	121.87 (15)	C16—C15—H15	120.1
C6—C7—C10	114.06 (14)	C15—C16—C11	122.12 (18)
C7—C8—S1	125.94 (14)	C15—C16—Cl1	118.14 (16)
C7—C8—H8	117.0	C11—C16—Cl1	119.73 (15)
S1—C8—H8	117.0	C8—S1—C9	99.34 (8)
C5—C9—C1	119.06 (19)		
			
C9—C1—C2—C3	0.9 (4)	C2—C1—C9—S1	175.81 (18)
C1—C2—C3—C4	−0.1 (4)	C8—C7—C10—C11	119.48 (19)
C2—C3—C4—C5	−0.2 (4)	C6—C7—C10—C11	−65.55 (19)
C3—C4—C5—C9	−0.4 (3)	C7—C10—C11—C12	96.9 (2)
C3—C4—C5—N1	−176.6 (2)	C7—C10—C11—C16	−81.8 (2)
C4—C5—N1—C6	−131.9 (2)	C16—C11—C12—C13	0.9 (3)
C9—C5—N1—C6	52.0 (3)	C10—C11—C12—C13	−177.81 (19)
C5—N1—C6—O1	169.22 (17)	C11—C12—C13—C14	−0.7 (3)
C5—N1—C6—C7	−9.9 (3)	C12—C13—C14—C15	−0.4 (4)
O1—C6—C7—C8	136.80 (19)	C13—C14—C15—C16	1.3 (3)
N1—C6—C7—C8	−44.0 (3)	C14—C15—C16—C11	−1.1 (3)
O1—C6—C7—C10	−38.0 (2)	C14—C15—C16—Cl1	−179.98 (17)
N1—C6—C7—C10	141.13 (17)	C12—C11—C16—C15	0.0 (3)
C6—C7—C8—S1	7.7 (3)	C10—C11—C16—C15	178.67 (17)
C10—C7—C8—S1	−177.85 (14)	C12—C11—C16—Cl1	178.87 (14)
C4—C5—C9—C1	1.2 (3)	C10—C11—C16—Cl1	−2.4 (2)
N1—C5—C9—C1	177.26 (17)	C7—C8—S1—C9	55.28 (19)
C4—C5—C9—S1	−176.03 (15)	C5—C9—S1—C8	−59.33 (16)
N1—C5—C9—S1	0.0 (2)	C1—C9—S1—C8	123.43 (16)
C2—C1—C9—C5	−1.5 (3)		

### Hydrogen-bond geometry (Å, º)

**Table:**

*D*—H···*A*	*D*—H	H···*A*	*D*···*A*	*D*—H···*A*
N1—H1*A*···O1^i^	0.82 (2)	2.08 (2)	2.8911 (19)	174 (3)

Symmetry code: (i) −*x*+1, −*y*+2, −*z*+2.

## References

[bb1] Bernstein, J., Davis, R. E., Shimoni, L. & Chang, N.-L. (1995). *Angew. Chem. Int. Ed. Engl.* **34**, 1555–1573.

[bb2] Bruker (2008). *APEX2*, *SAINT* and *SADABS* Bruker AXS Inc., Madison, Wisconsin, USA.

[bb3] Farrugia, L. J. (1997). *J. Appl. Cryst.* **30**, 565.

[bb4] Huang, Z.-H., Chu, Y. & Ye, D.-Y. (2011). *Acta Cryst.* E**67**, o168. 10.1107/S1600536810052098 PMC3050135 21522675

[bb5] Metys, J., Metysova, J. & Votava, Z. (1965). *Acta Biol. Med. Ger.* **15**, 871–873. 4160911

[bb6] Rajsner, M., Protiva, M. & Metysova, J. (1971). Czech. Patent Appl. CS 143737.

[bb7] Sheldrick, G. M. (2008). *Acta Cryst.* A**64**, 112–122. 10.1107/S0108767307043930 18156677

[bb8] Spek, A. L. (2009). *Acta Cryst.* D**65**, 148–155. 10.1107/S090744490804362X PMC2631630 19171970

[bb9] Tomascovic, L. L., Arneri, R. S., Brundic, A. H., Nagl, A., Mintas, M. & Sandtrom, J. (2000). *Helv. Chim. Acta*, **83**, 479–493.

